# Postpartum hemorrhage with associated placenta previa in a kidney transplant recipient: A case report

**DOI:** 10.1016/j.ijscr.2023.109109

**Published:** 2023-12-08

**Authors:** Toshinao Suzuki, Takahiro Sugiura, Junko Okazaki, Hiroaki Kimura

**Affiliations:** a Department of Anesthesiology, Kimitsu Chuo Hospital, 1010 Sakurai, Kisarazu, Chiba 292-8535, Japan; b Department of Obstetrics and Gynecology, Kimitsu Chuo Hospital, 1010 Sakurai, Kisarazu, Chiba 292-8535, Japan

**Keywords:** Postpartum hemorrhage, Placenta previa, Kidney transplantation, Uterine artery embolization, Balloon occlusion, Case report

## Abstract

**Introduction:**

The efficacy and safety of uterine artery embolization (UAE) and prophylactic resuscitative endovascular balloon occlusion of the aorta (REBOA) against postpartum hemorrhage (PPH) in pregnant women after kidney transplantation have not been reported. Here, we describe a case of PPH associated with placenta previa in pregnancy following kidney transplantation, which was managed with UAE and prophylactic REBOA.

**Case presentation:**

A 35-year-old, gravida 2, para 1 woman with total placenta previa presented with vaginal bleeding (460 mL) at 33 weeks and 3 days of gestation. Previously, she underwent a living-donor kidney transplantation for IgA nephropathy, and the renal artery of the transplanted kidney was anastomosed with the right internal iliac artery. An emergency cesarean section with prophylactic REBOA was performed under general anesthesia. A balloon catheter was introduced via the left femoral artery and positioned above the aortic bifurcation (Aortic zone 3). Upon confirming fetal delivery, the balloon was immediately inflated, and the total aortic occlusion time was 20 min. However, following aortic balloon deflation, atonic bleeding continued despite Bakri balloon usage and uterotonic drug administration. Subsequently, UAE was performed for the refractory PPH, the left uterine artery was embolized using a gelatin sponge, and hemostasis was successfully achieved. The patient recovered uneventfully and was discharged on postoperative day 7.

**Discussion and conclusion:**

In pregnancies following kidney transplantation, prophylactic REBOA controls bleeding; however, it decreases blood flow to the transplanted kidney. Furthermore, uterine nutrient vasculature alterations are observed, necessitating a thorough understanding of the uterine artery supply pathways during UAE.

## Highlights

• Postpartum hemorrhage after kidney transplantation can be managed endovascularly.
• Kidney transplantation to the pelvis alters uterine blood flow dominance.
• Aortic balloon occlusion may reduce blood flow to the transplanted kidney.
• Embolization effectively manages postpartum hemorrhage in transplanted kidneys.

## 1 Introduction

The number of kidney transplant recipients is increasing yearly [1]. Despite the increased risks of complications for mother and child, pregnancy can be achieved after kidney transplantation [[2], [3], [4]]. Postpartum hemorrhage (PPH) is a major contributor to perinatal mortality [5]; however, reports of PPH associated with pregnancies following kidney transplantation are rare, and data regarding its incidence and mortality rate remain unclear. The efficacy of uterine artery embolization (UAE) as a hemostatic method for intractable PPH [6] and prophylactic resuscitative endovascular balloon occlusion of the aorta (REBOA) for mitigating bleeding during cesarean section [7,8] has been documented. UAE and prophylactic REBOA are increasingly used to manage PPH. However, the safety and efficacy of UAE and prophylactic REBOA in pregnancies after kidney transplantation have not yet been established. In this novel report, we present a case of PPH associated with placenta previa during pregnancy following kidney transplantation for IgA nephropathy, which was managed with UAE and prophylactic REBOA. This case report adheres to the SCARE 2020 criteria [9].

## 2 Presentation of case

A 35-year-old woman, gravida 2, para 1, was scheduled for elective cesarean section after 36 weeks of gestation due to total placenta previa. The patient had previously undergone living-donor kidney transplantation at another hospital 11 years ago due to end-stage renal failure caused by IgA nephropathy and was treated daily with the following triple-immunosuppressant regimen: tacrolimus (1.5 mg), azathioprine (100 mg), and prednisolone (5 mg). Laboratory test results revealed a serum creatinine level of 0.91 mg/dL, a hemoglobin level of 10.0 g/dL, and no evidence of coagulopathy (platelets, 162,000/μL; prothrombin time, 10.3 s; activated partial thromboplastin time, 26.8 s). At 33 weeks and 3 days of gestation, 460 mL of vaginal bleeding was observed, leading to an emergency cesarean section with prophylactic REBOA. Before the cesarean section, a 7-Fr REBOA device (Rescue Balloon ER®, Tokai Medical Products, Aichi, Japan) was introduced and positioned with an 8-Fr sheath in the left femoral artery using sonography. The balloon placement above the aortic bifurcation (Aortic zone 3) was confirmed using fluoroscopy. Subsequently, general anesthesia was administered, and the cesarean section was performed. The REBOA was inflated immediately upon confirmation of fetal delivery. Total aortic occlusion via REBOA was confirmed through the disappearance of arterial pressure in the femoral sheath used for REBOA. The placenta was delivered successfully without complications. Finally, oxytocin (intramuscular and intravenous administration of 5 and 20 units, respectively) and 1 g tranexamic acid were administered. Due to persistent atonic bleeding, compression was applied using a Bakri balloon. REBOA was deflated after the Bakri balloon was inflated (aortic occlusion time was 20 min). Although the Bakri balloon initially controlled the bleeding, the bleeding persisted due to complicated coagulopathy with low fibrinogen levels (fibrinogen, 113.1 mg/dL), leading to the decision to perform endovascular embolization. After performing a temporary abdominal closure, the patient was transported to the angiography room, where endovascular embolization was performed. The balloon occlusion catheter was removed, and angiography was performed through the left femoral sheath.

**Fig. 1:**
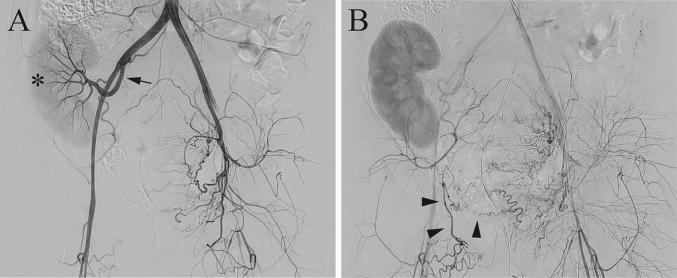
Pelvic aortography before embolization. A) Pelvic aortography reveals the right-sided location of the kidney within the pelvis (asterisk) while visualizing blood flow from the right internal iliac artery (arrow). B) In the later phase of pelvic angiography, the right external iliac artery provides blood flow to the right uterine artery (arrowhead).

**Fig. 2:**
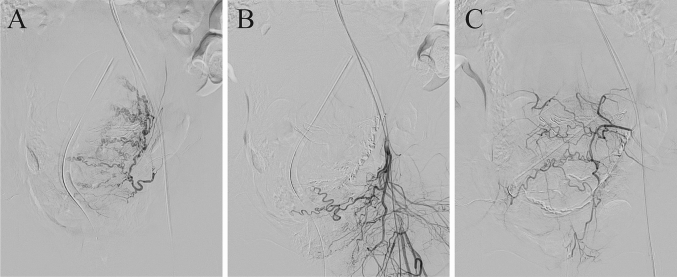
Angiography reveals the uterus being perfused by a branch vessel stemming from the left internal iliac artery. A) Selective angiography from the left uterine artery. B) Angiography conducted after the embolization of the left uterine artery confirms the presence of blood flow to the uterus originating from a branch of the inferior gluteal artery. C) Selective angiography of the lateral sacral artery additionally confirms the presence of blood flow to the uterus.

Pelvic angiography revealed that the left internal iliac artery provided blood flow to the transplanted renal artery, whereas collateral circulation from the right external iliac artery supplied blood to the right uterine artery (Fig. 1). The left uterine artery was selectively catheterized and embolized with a gelatin sponge (Fig. 2A). Additionally, the feeding vessels connected to the uterus, which originated from branches of the inferior gluteal and lateral sacral arteries (Fig. 2B and C), were meticulously identified and subsequently embolized. Following embolization, external bleeding ceased, and the procedure was concluded with embolization on the left side only. The newborn had a birth weight of 2246 g and Apgar scores of 1, 8, and 10 at 1, 5, and 7 min, respectively. The delivery of the fetus and placenta occurred at 4 and 5 min, respectively, following the initiation of surgery. Subsequently, myometrial suturing was performed, and temporary closure of the abdomen required 41 min from the outset. Following this, the patient was transferred to the angiography room. Vascular embolization was accomplished within 43 min, and an additional 54 min were necessary to verify hemostasis and perform wound closure in the operating room. The total blood loss was 4305 mL, and intraoperative blood transfusions included 12, 10, and 12 units of red blood cells, fresh frozen plasma, and cryoprecipitate, respectively. Finally, a total of 45 units of oxytocin and 0.2 mg of methylergometrine were administered intraoperatively. The histological examination of the placenta revealed no evidence of placenta accreta spectrum. The postoperative course was uneventful, with no decline in creatinine levels. The patient was discharged on day 7 of hospitalization. Following the preference of the patient, hysterectomy was successfully avoided, thereby preserving the possibility of fertility. Subsequently, the newborn was admitted to the general ward, and no postnatal complications occurred.

## 3 Discussion

PPH is the leading cause of maternal mortality worldwide [5]. Nevertheless, data pertaining to the incidence and mortality rates of PPH in pregnancies following kidney transplantation are lacking despite the increasing number of kidney transplantation cases [1]. Therefore, PPH should be considered in pregnancies after kidney transplantation. Here, we described a case of PPH associated with placenta previa in a kidney transplant recipient who was successfully treated with prophylactic REBOA and endovascular embolization.

REBOA is a minimally invasive procedure involving the introduction of a balloon occlusion catheter into the aorta to achieve endovascular aortic occlusion [7,10]. Recently, prophylactic REBOA has been utilized to reduce bleeding in PPH cases, and its effectiveness has been investigated [7,8,11]. However, awareness of the risks associated with REBOA, such as ischemia-reperfusion-related complications, including acute kidney injury, limb ischemia, spinal cord ischemia, and visceral organ dysfunction, is necessary [12,13].

In patients who have undergone kidney transplantation, performing REBOA requires an increased awareness of atypical pelvic blood flow dynamics. When the internal iliac artery is ligated, blood flow within the uterine artery is sustained through collateral channels originating from the external iliac and ovarian arteries [14,15]. Hence, even when the internal iliac artery is treated due to kidney transplantation, REBOA blocks the blood flow within the external iliac artery, potentially aiding blood flow control. However, when prophylactic REBOA is conventionally performed for PPH, it is frequently positioned below the renal artery. In cases such as the one described here, where kidney transplantation involves anastomosis between the internal iliac and renal arteries, the placement of an occlusion balloon catheter may inadvertently obstruct renal blood flow. Considering circulatory conditions, it is imperative to minimize ischemic time whenever possible to preserve renal blood flow. When employing REBOA in the preparation of kidney transplant patients to mitigate posttransplant hemorrhage, it is vital to develop a management strategy that not only minimizes blood loss but also circumvents any unwarranted reduction in renal blood flow, thereby averting the deterioration of renal function. In cases involving REBOA in renal transplant recipients, it is imperative to recognize that balloon occlusion at the level of the aortic bifurcation (Aortic zone 3) can result in decreased renal blood flow.

REBOA serves as an adjunct and does not independently confer hemostatic effects. UAE proves effective in intractable PPH treatment [6]. UAE has become increasingly prevalent and plays a pivotal role in PPH management owing to its numerous advantages, including effectiveness, safety, minimal invasiveness, paucity of complications, and preservation of fertility [6]. However, the effectiveness and safety of UAE for PPH during pregnancy after kidney transplantation remain uncertain. The utilization of the internal iliac artery is a common practice [16]. Consequently, the uterine artery, a branch of the internal iliac artery, undergoes alterations in its supply source, which affects the blood supply to the uterus. When the internal iliac artery is involved in renal transplantation, collateral pathways, such as the external iliac artery, may contribute to the supply to the uterine artery [15]. In this case, besides the blood supply originating from the left external iliac artery to the left uterine artery, blood flow to the uterus was also confirmed from branches of the right internal iliac artery, specifically the lateral sacral and inferior gluteal arteries. Thus, following kidney transplantation in pregnant women, the vascular supply to the uterus may be altered. In this case, we performed an emergency cesarean section without adequate preoperative evaluation. However, in cases of cesarean section with a high risk of impending hemorrhage, it is imperative to assess the anatomical integrity of the uterine nutrient vessels in advance.

## 4 Conclusion

We report a case of PPH associated with placenta previa in a pregnant woman following kidney transplantation, managed successfully with prophylactic REBOA and UAE. Hysterectomy was avoided, and no ischemic complications, such as worsening renal function, were observed. These findings indicate that after renal transplantation in pregnant women, prophylactic REBOA is beneficial in controlling bleeding; however, it decreases blood flow to the transplanted kidney. Furthermore, alterations in the uterine nutrient vasculature were observed, highlighting the importance of a thorough understanding of the uterine artery supply pathways during UAE.

## Consent for publication

Written informed consent was obtained from the patient for publication and any accompanying images. A copy of the written consent is available for review by the Editor-in-Chief of this journal on request.

## Ethical approval

The requirement for ethical approval was waived by our institution. The patient provided informed consent for the publication of medical information in this case report.

## Funding

This study did not receive any specific grants from funding agencies in the public, commercial, or not-for-profit sectors.

## CRediT authorship contribution statement

Toshinao Suzuki: Conceptualization, Writing - Original Draft. Takahiro Sugiura: Writing - Review & Editing. Junko Okazaki: Writing - Review & Editing. Hiroaki Kimura: Writing - Review & Editing. All the authors have read and approved the final version of the manuscript.

## Guarantor

Toshinao Suzuki.

## Research registration number

Not applicable.

## Declaration of competing interest

The authors declare that they have no competing interests.

## Data Availability

Not applicable.
